# Isolated, traumatic posterior dislocation of the radial head in an adult: A new case treated conservatively

**DOI:** 10.4103/0974-2700.70767

**Published:** 2010

**Authors:** Abdelhalim El Ibrahimi, Mohammed Shimi, Abdelkrim Daoudi, Abdelmajid Elmrini

**Affiliations:** Department of Orthopaedic Surgery B4, UH of Fez, Morocco

Sir

**Figure 1:**
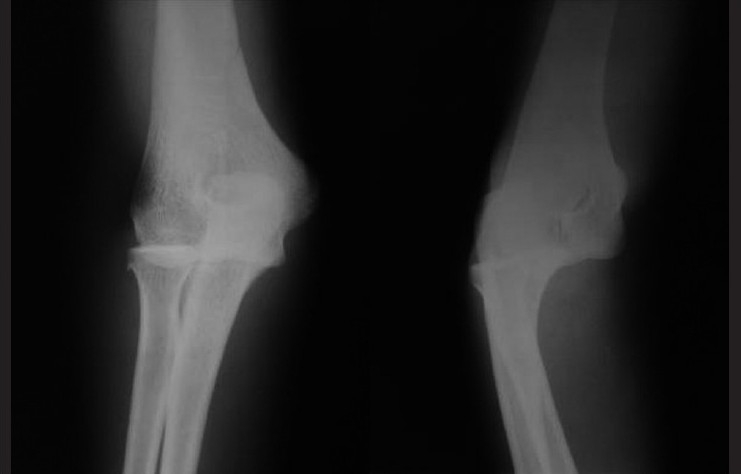
Radiographs of the elbow showing a posterior dislocated radial head

**Figure 2:**
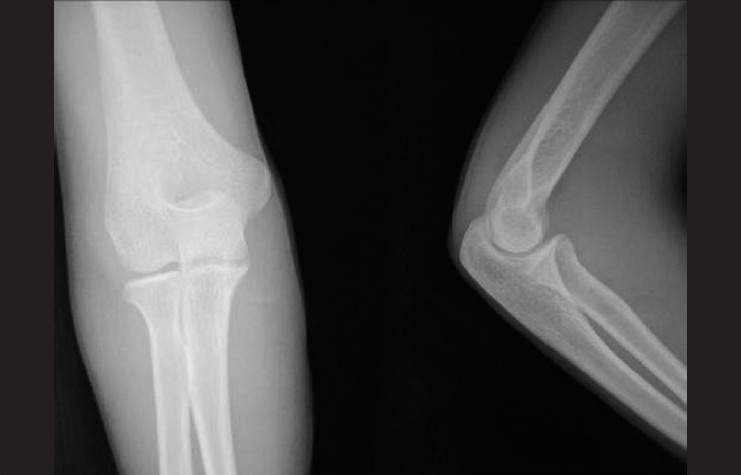
Postreduction radiographs of the elbow showing the radial head in reduced position

In adults, isolated radial head dislocation is an extremely rare injury. Earlier reports of cases of acute radial head dislocation in adults have been associated with either fracture of ulna or when isolated, restricted to children or adolescents. A 26-year-old male reported to the emergency department with complaints of pain and restricted range of motion in the right elbow following a fall off a motorcycle on his dominant hand. He had swelling obvious on the lateral and posterior aspect of the elbow. The elbow was held in flexion and partial supination. Tenderness was present laterally at the elbow, and the radial head was palpable posteriorly. There was no swelling or tenderness over the ulna. Neurovascular examination of the upper limb did not reveal any abnormality. There was no tenderness over the forearm or distal radioulnar joint. Radiographs showed a posterior dislocation of the radial head. No abnormality of the ulna was noted [Figure 1]. Under general anesthesia, gentle traction, pronation, and direct pressure over the radial head were used to reduce the dislocation. Postreduction, the elbow was found to be stable [Figure 2]. Immobilization was done in a long-arm cast for 4 weeks. At final follow-up, the patient had recovered complete range of motion.

Isolated dislocation of the radial head without concomitant ulnar fracture or humeroulnar subluxation in adults is a rare injury.[1–5] Most cases appear to be in children.[6] Heidt and Sterne, in 1982, were the first to describe this injury.[7] Only 22 cases have been reported in adults in the literature. It has been predominantly posterior.[8]

The mechanism leading to an isolated radial dislocation has been variously described. Most authors describe an indirect mechanism. The proximal radioulnar joint is most stable in supination: in this position, the contact between radius and ulna is maximal and the interosseous membrane, the annular ligament, and the anterior fibres of the quadrate ligament are all taut, thus drawing the radial head snugly against its notch in the ulna. Cadaveric studies have shown that posterior dislocation of the radial head cannot occur without the rupture of the annular ligament; in addition, partial tear of the quadrate ligament and the proximal interosseous membrane takes place.[9]

We speculate the mechanism in our patient to be a hyperextension of the elbow with forearm in prone position leading to a posterior dislocation of the radial head.

Typical clinical presentation is maintenance of flexion and extension following the injury, but complete loss of pronation and supination.

There are no guidelines for treatment. In the literature, 15 cases were treated conservatively with no recurrence. This is an additional case treated conservatively. If diagnosed on time, acute cases can be reduced by an easy external manipulation, and the functional outcome seems to be good postreduction. Only one irreducible dislocation has been previously reported in an adult.[10]

Most authors propose immobilization of the elbow in flexion and supination in a plaster cast,[6] whereas Bonatus *et al*.,[2] Negi *et al*.,[1] and Obert *et al*.[8] immobilized their cases in flexion and pronation. The period of immobilization varied from 10 days[8] to 4 weeks for readaptation.

If missed or neglected, an open reduction must to be done with either an annular ligament reconstruction[3] or a radial head excision;[7] in these conditions, the results were poor with a restriction of forearm supination and pronation and significant risk of degenerative arthritis of the elbow and the distal radioulnar joints.

An inability to pronate/supinate while able to flex the elbow should suggest posterior radial head dislocation. The diagnosis may be easily missed on radiographs and, therefore, require a high index of suspicion. Early reduction is important in order to avoid the necessity for excision of the head of radius and its attendant complications.
